# Default Sex and Single Gene Sex Determination in Dioecious Plants

**DOI:** 10.3389/fpls.2020.01162

**Published:** 2020-07-29

**Authors:** Quentin Cronk, Niels A. Müller

**Affiliations:** ^1^ Department of Botany and Biodiversity Research Centre, University of British Columbia, Vancouver, BC, Canada; ^2^ Thünen Institute of Forest Genetics, Grosshansdorf, Germany

**Keywords:** dioecy, monoecy, *Populus*, *ARR17*, *Diospyros*, *OGI*, *SRY*, MADS-box

## Abstract

A well-established hypothesis for the evolution of dioecy involves two genes linked at a sex-determining region (SDR). Recently there has been increased interest in possible single gene sex determination. Work in *Populus* has finally provided direct experimental evidence for single gene sex determination in plants using CRISPR-Cas9 to knock out a single gene and convert individuals from female to male. In poplar, the feminizing factor *popARR17* acts as a “master regulator”, analogous to the mammalian masculinizing factor SRY. The production of fully functional males from females by a simple single gene knockout is experimental evidence that an antagonistic male-determining factor does not exist in *Populus*. Mammals have a “default sex” (female), as do poplar trees (*Populus*), although the default sex in poplars is male. The occurrence of single gene sex determination with a default sex may be much commoner in plants than hitherto expected, especially when dioecy evolved *via* monoecy. The master regulator does not even need to be at the SDR (although it may be). In most poplars the feminizing factor *popARR17* is not at the SDR, but instead a negative regulator of it. So far there is little information on how high-level regulators are connected to floral phenotype. A model is presented of how sex-determining genes could lead to different floral morphologies *via* MADS-box floral developmental genes.

## Introduction: the Two-Gene and Single-Gene Models

The “two-gene model” for the evolution of dioecy put forward in 1978 has been enormously influential, the original paper being cited over 1,000 times (Charlesworth and Charlesworth, 1978). This model posits that two mutations (most probably in two separate genes, a male-sterility factor and a female-sterility factor) are likely to be involved in the evolution of dioecy, and further that these two genes are usually brought together in linkage. This model was developed in the context of the evolution of dioecy through the gynodioecy pathway, and in this context it is well supported. *Silene*, for instance, has iconic status as a model system for the two-gene system (*e.g.* Westergaard, 1958; Kazama et al., 2016).

However, dioecy also evolves through the monoecy pathway, and here the model may be less useful. For instance, in a monoecious plant it is likely that there has been a mutation to allow spatiotemporal factors (such as hormone gradients) to interact with development to produce male or female flowers, depending on the position within the plant or on developmental timing. Floral developmental pathways in monoecy could therefore evolve to be under the overall control of a single high-level regulator with two states (on *vs* off) determining the two phenotypes. If this high-level regulator is segregating in the population (present *vs* absent), then dioecy, involving only a single regulatory gene, could readily evolve from monoecy. This is the alternative “single-gene model” (Renner, 2016) which this perspective argues is likely to be most commonly involved in the evolution of dioecy through the monoecy pathway.

Two final points should be mentioned here: first, discussion of “single-gene sex determination” or “two-gene sex determination” refers not to the total numbers of genes involved in sexual differentiation: the pathway that leads to the suppression of stamens or carpels may, of course, have multiple genes involved. Rather it refers to the numbers of genes needed for the segregation of sex at the sex-determining region (SDR) of the genome. Secondly, the SDR may be a genomic block subject to recombination suppression, in which case the chromosome may be characterized as a sex chromosome, and the other chromosomes as autosomes. However, the sex chromosome/autosome distinction is complicated if the region of recombination suppression is small (as in many plants) or even non-existent. In the pufferfish, *Takifugu rubripes*, sex determination is effected by one single nucleotide polymorphism (Kamiya et al., 2012). Here we refer to regions of the genome as sex-determining (SDR) or “autosomal” (outside the SDR).

## Recent Results on the Genetic and Molecular Basis of Sex Determination

Very rapid progress is being made in understanding the genetic and molecular basis of dioecy in flowering plants with important papers in a diverse array of systems including papaya (*Carica:* Liu et al., 2004) and more recently, kiwifruit (*Actinidia*: *e.g.* Akagi et al., 2019), persimmon (*Diospyros:* Akagi et al., 2020), date palm (*Phoenix:* Torres et al., 2018), grape (*Vitis*: Massonnet et al., 2020), asparagus (*Asparagus:* Harkess et al., 2017), and poplar (*Populus:* Geraldes et al., 2015; Müller et al., 2020). The first five of these are fruit crops in which dioecy has obvious economic importance in fruit production. Asparagus and poplar are vegetable and tree crops, respectively, and are also important in the role they have in shedding light on dioecy. So far, molecular work in most economically important species has provided support for the two-gene model, for instance in kiwifruit (Akagi et al., 2019), *Vitis* (Massonnet et al., 2020), *Phoenix* (Torres et al., 2018), and asparagus (Harkess et al., 2020). However, in two other systems for which dioecy is well characterized at the molecular level, persimmon and poplar, the results seem to point at a one-gene model at the SDR.

In *Diospyros lotus* (Akagi et al., 2014), there is an autosomal feminizing gene *MeGI*, giving rise to females. Males are produced when a Y-specific suppressor, *OGI*, inhibits *MeGI* expression. Notably, transcriptional regulation of *MeGI* is also involved in the control of sex expression in monoecious persimmon (*D. kaki*: Akagi et al., 2016).

**Figure 1:**
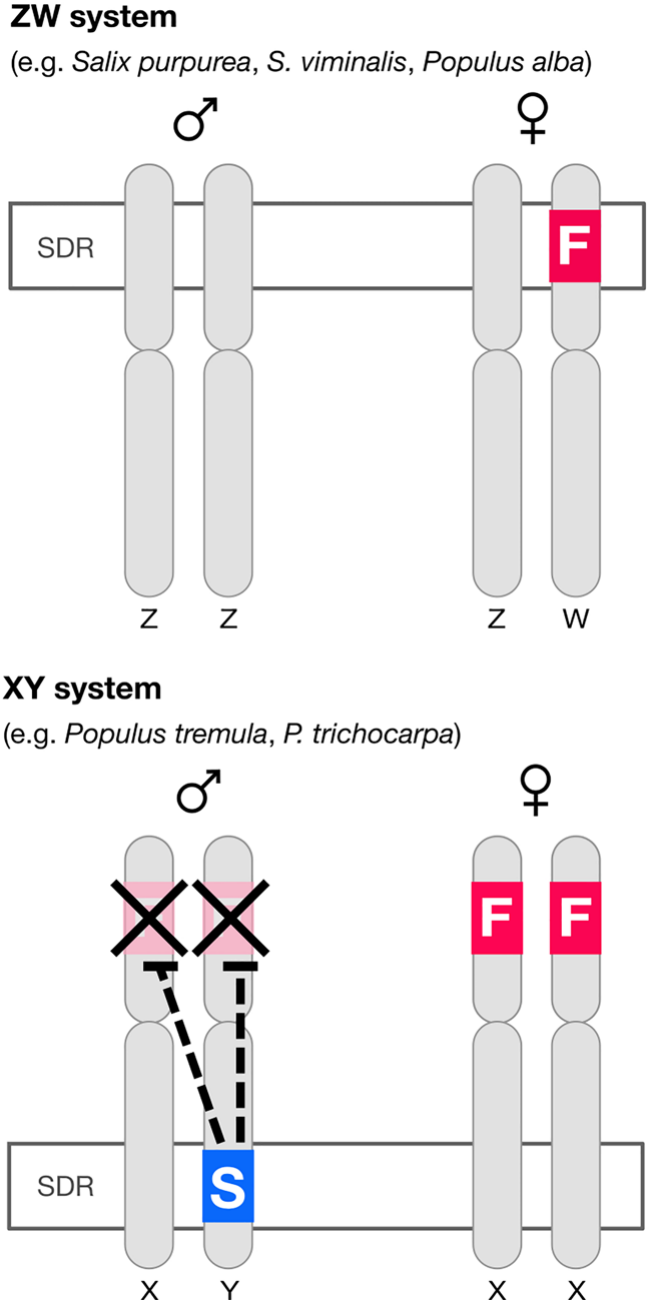
Diagram of the architecture of sex determination in willow and poplar. In at least some willow and one poplar species (*P. alba*) the default developmental pathway is male but a feminizing factor F (the homolog of the cytokinin response regulator *ARR17*) segregates, giving a ZW sex determination system. In most poplar species the default developmental pattern is again male, but both sexes have a copy of the feminizing factor. A “suppressor of F” factor (S) segregates, giving an XY system of sex determination. The “suppressor of F” consists of an *ARR17* pseudogene inverted repeat (*ΨARR17-IR*). This structure leads to the production of sRNA and thence to RNA-directed DNA methylation (RdDM) silencing *ARR17* (Müller et al., 2020). The feminizing factor *ARR17* is therefore analogous to the masculinizing factor SRY in mammals (except sex-reversed).

In poplar (Müller et al., 2020) there is an autosomal (*i.e.* not associated with the sex-determining region, or SDR) feminizing gene *popARR17* (the poplar homolog of Arabidopsis Response Regulator 17). There is also, critically, a Y-specific suppressor of *ARR17* at the SDR (*Ψ*ARR17-IR) (**Figure 1**). In poplar, *Ψ*ARR17-IR is an inverted repeat of the target gene that forms hairpin RNA, generating sRNA and silences *popARR17* by RNA-directed DNA methylation (RdDM) (**Figure 1**). In one species of poplar (*Populus alba*: Müller et al., 2020) and apparently in several willow species (*Salix:* Almeida et al., 2020; Zhou et al., 2020) the same basic sex-determining system appears to be used (discussed in Müller et al., 2020), except here the “master regulator” *ARR17* is hemizygous at the SDR on the W chromosome: it is inferred that females develop when it is present (ZW), males when it is absent (ZZ), implying that males are the “default sex” (**Figure 1**).

## The Concept of a Default Sex and a “Master Regulator”

The sex-determination system of mammals (specifically eutherians and marsupials) is a well-worked out single-gene system with a master regulator (SRY) and a default sex (female). The mammalian system now appears to provide a surprisingly useful analogous system for sex determination in some plants.

Even before the discovery of SRY, the notion of female as the mammalian “default sex” was established experimentally. An inferred gene, testes determining factor (TDF) was shown to act dominantly in mammalian development. If testes are removed from young XY embryos, they develop as females. Whereas if ovaries are removed from young XX embryos, they still develop as females (Jost, 1970), implying that a specific signal was required for male development, but not for female development. For this reason females were considered to be the default sex (Graves, 2017). Later, the gene ‘Sex-determining Region on the Y’ or *SRY* was discovered (Sinclair et al., 1990) which fitted the requirements of the TDF master regulator that could divert default female development into male development. If SRY is mutated to be non-functional, female development ensues despite the presence of a Y chromosome (Berta et al., 1990). Conversely if SRY is introduced transgenically into XX females, males develop (Koopman et al., 1991). However, it should be noted that mammalian sex determination is complex (*e.g.* Zhao et al., 2017) and monotremes are very different (Veyrunes et al., 2008).

What is the equivalent in persimmon, poplar and willow? In *Populus alba* and in ZW willow species, *ARR17* is present only on the W chromosome, apparently as a dominant feminizer, giving rise to ZW females, and in its absence, to default ZZ males. The parallels to SRY are obvious (except sex-reversed) and imply that *ARR17* is the master regulator and male the default sex. This is complicated in other poplars however, where the *ARR17* “master regulator” is autosomal and is toggled on/off by a Y-specific suppressor at the SDR. But it is not helpful to consider the suppressor the master regulator: the fundamental regulation by *ARR17* remains the same, as does the default sex (male). It is only the regulation of ARR17 that differs (toggled on/off by a repressor in XY poplar and regulated by the presence/absence in ZW willow).

The first experimental evidence in dioecious plants of both the existence of a master regulator (*ARR17*) and a default sex (male) was obtained in poplar by CRISPR-Cas9 knock-out of *ARR17* (Müller et al., 2020) which converted XX females to males, reminiscent of SRY knock-outs in mammals (but sex-reversed). The fact that in poplar a *single gene is necessary and sufficient* for female development, and without it males develop, is powerful evidence for a single master regulator and a default sex. The CRISPR result shows that an antagonistic male determining factor is not necessary in poplar. Instead the system is driven by a single gene, the feminizing factor *popARR17*.

The situation in *Diospyros* seems very similar and can be interpreted as an autosomal feminizing master regulator (*MeGI*) capable of redirecting default male development to female and toggled on–off by the presence of a suppressor at the SDR. We thus have two potential models for the molecular control of dioecy: (1) a segregating hemizygous single gene master regulator at the SDR (*Salix, Populus alba*) or (2) an autosomal single gene master regulator, toggled on–off by a segregating suppressor at the SDR (*Diospyros, Populus*).

Monoecious plant species also need to control unisexual flower development, only in different parts of the same plant instead of different individuals. Interestingly, transcriptional regulation of a feminizing master regulator appears to control this differential sexual development in diverse monoecious species as well. In monoecious maize and cucumber, for example, the genes *Silkless* and *ACS11*, respectively, trigger female development, and mutations lead to purely male plants (androecy) (Jones, 1934; Boualem et al., 2015). It is simple to imagine an adoption of this mechanism in the evolution of dioecy by limiting expression of the feminizing master regulator to female individuals instead of creating an expression gradient within one plant. As the unisexual separation of floral developmental pathways has already occurred in the monoecious ancestor, the evolution of dioecy from monoecy may be a simple matter of separating pre-existing epistatic regulatory architecture to different individuals, which may be accomplished by a single segregating gene. For this reason, we might expect the evolution of single gene-sex determination systems to be associated with monoecious lineages. Willow and poplar appear to be examples of this, as the family Salicaceae (to which they belong) is largely composed of monoecious or “polygamous” (*i.e.* mixing hermaphrodite and unisexual flowers) species (Cronk, 2005; Cronk et al., 2015).

## Enter the Mads-Box Genes

Despite the rapid advances in understanding the high-level molecular mechanisms for dioecy, there has been little progress on how those high-level regulators are connected to phenotypes through gene regulatory cascades. There is likely to be a fundamental difference in the development between dioecious plants in which organs of the opposite sex are deleted and those where they are vestigial. So, in dioecious members of the Rosaceae it is common to find vestigial stamens in female flowers, whereas in the poplar there is no sign whatever of stamens in female flowers (apparently at any stage of development): they are cleanly deleted. Floral MADS-box genes are required to specify the identity of floral organ types, according to the well-known ABC model (Litt and Kramer, 2010). If the required combinations of MADS-box genes are not expressed, the relevant organs will not form. Therefore MADS-box genes are candidates for clean deletion of stamens or carpels. However, in cases of dioecy where floral organs are vestigial, it is unlikely that MADS-box genes are involved as they must be expressed correctly if the relevant organs (even if vestigial) are formed. Instead it is likely that genes downstream of the MADS-box gene pathway are blocking development of those organs once formed.

**Figure 2:**
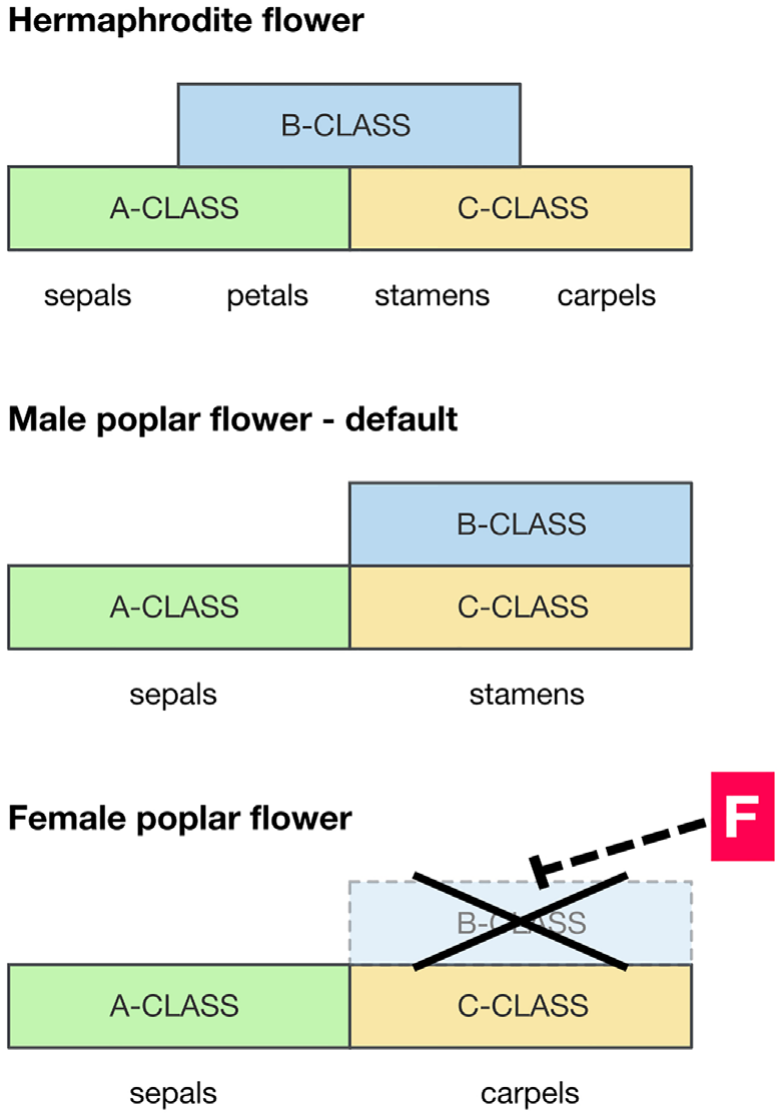
Hypothetical scenario for the developmental pathway leading to male and female floral development in poplar and willow. Diagrams of the expression domains of MADS-box floral developmental genes relative to floral organ development. Top panel: pattern typical of a hermaphrodite eudicot flower (*e.g.* *Arabidopsis*), for reference. Bottom panels: suggested MADS-box gene expression domains in the formation of male and female poplar flowers. Where the B-class MADS-box genes *PISTILLATA* and *APETALA3* (*PI/AP3*) are expressed, along with C-class genes, stamens develop. A wide expression domain of *PI/AP3* is therefore expected in males, and this is likely to be the default in both sexes. However, in females the presence of a feminizing factor F downregulates *PI/AP3* diverting development to a female developmental pathway.

*PISTILLATA* and *APETALA3* (*PI/AP3*) are a pair of MADS-box genes required for the specification of stamens (class-B MADS-box genes), and where they are expressed carpels do not form (**Figure 2**). Constitutive overexpression of *PI/AP3* is therefore expected to produce stamen-only flowers as seen in male poplars (Cronk et al., 2020). The action of the feminizing factor ARR17 therefore could be to locally block the expression of *PI/AP3*, inhibiting stamens and allowing carpels to form (as in female flowers). A hypothetical model of this is presented in **Figure 2**. However, the pathway between ARR17 and PI/AP3 (if it exists) is completely unknown. This should be a target for future research. ARR17 is a response regulator implicated in regulation of the cytokinin pathway, but its mode of action in floral development at present remains elusive. In considering the potential involvement of *PI* it is of significance that the sex determinant MeGI in persimmon (which acts as a repressor of the androecium) functions *via* its ability to regulate *PI* during early androecium development (Yang et al., 2019). In addition, *Nicotiana tabacum* transgenic for MeGI showed significant downregulation of *PI* (Akagi et al., 2020).

## Conclusions

Current molecular results on sex-regulation in *Populu*s and *Diospyro*s provide evidence for two related pathways, neither of which require two linked genes at the SDR. The first involves an autosomal master regulator (*ARR17*, *MeGI*), toggled on–off by a suppressor at the SDR (*ΨARR17-IR*, *OGI*). The second involves a hemizygous master regulator at the SDR (*ARR17* in *P. alba* and *Salix*). Many more examples will need to be worked through in other plants before we can determine with certainty whether this is the norm, or whether the model of two linked loci at the SDR (as seems to be the case in *Asparagus* or kiwifruit) is more usual. More work on other systems is therefore a priority. It may be, however, that a single gene at the SDR is all that is needed to regulate sex, and this will be much more common than currently thought (especially in the monoecy–dioecy pathway where floral dimorphism has already evolved).

Furthermore, the pathway connecting these high-level regulators to floral phenotype is largely unknown, and work in this area is urgently required if we are to fully understand dioecy. In this regard, attention is drawn to two very different developmental outcomes: deleted organs *vs*. vestigial organs. The first is likely to involve changes in floral MADS-box gene expression (especially *PI/AP3*), whereas the second is likely to involve genes involved in organ growth and maturation downstream of the MADS-box gene pathway.

## Data Availability Statement

The original contributions presented in the study are published (see citations); further inquiries can be directed to the corresponding author.

## Author Contributions

QC and NM developed the ideas and wrote the manuscript.

## Funding

Work in the laboratory of QC is funded by the Natural Sciences and Engineering Research Council of Canada (NSERC). NM acknowledges the support of the Deutsche Forschungsgemeinschaft (DFG: MU 4357/1-1).

## Conflict of Interest

The authors declare that the research was conducted in the absence of any commercial or financial relationships that could be construed as a potential conflict of interest.
